# Clinical features, etiology, and prognosis of hand knob stroke: a case series

**DOI:** 10.1186/s12883-022-02858-0

**Published:** 2022-09-02

**Authors:** Zhiyong Zhang, Xiaoxin Sun, Xinxiu Liu, Lei Wang, Rui Zhu

**Affiliations:** Department of Neurology, Beijing Geriatric Hospital, Beijing, China

**Keywords:** Hand knob stroke, Hand motor cortex, Hand paralysis, Stroke, Etiology

## Abstract

**Background:**

Hand knob stroke is a rare clinical disorder frequently misdiagnosed as peripheral neuropathy. The purpose of this study is to recognize this particular type of stroke by analyzing clinical features, etiology, and prognosis.

**Methods:**

We enrolled 19 patients with acute hand knob stroke in the Department of Neurology of the Beijing Geriatric Hospital from January 2018 to January 2022, and the clinical and imaging data of the patients during hospitalization and follow-up were collected and summarized.

**Results:**

Acute hand knob stroke accounted for 0.9% of all acute stroke, and ischemic stroke (17 cases, 89.5%) was more than hemorrhagic stroke (2 cases, 10.5%). All patients presented sudden contralateral hand paresis, 12 (63.2%) of them had only isolated hand paralysis, and the location of the lesion corresponded to different finger weakness. The cause of hand knob hemorrhage was hypertension, while the causes of hand knob infarction were mainly small-vessel occlusion (SVO) (35.3%) and large-artery atherosclerosis (LAA) (35.3%), and the rare causes include carotid artery dissection and carotid body tumor. After a median follow-up 13.5 months, the prognosis of 94.7% patients was good, and one patient (5.3%) had recurrent stroke.

**Conclusions:**

Hand knob stroke is a rare stroke with a good prognosis and a low stroke recurrence rate. Ischemic stroke is the predominant type and the main clinical manifestation is hand paresis. The cause of hand knob hemorrhage is hypertensive, while SVO and LAA are the main causes of hand knob infarction, but there are some rare etiologies.

## Background

The hand knob area is considered to be the cortical site of hand motor function, located in the precentral gyrus. It was described as an omega or epsilon-like shape in the axial plane and a hook-like shape in the sagittal plane by functional magnetic resonance imaging (MRI) study in 1997, and served as a reliable anatomical landmark for identifying the precentral gyrus [1]. Isolated hand palsy is mainly caused by peripheral neuropathy and rarely occurs in stroke [2]. Hand knob stroke can mimic radial and/or ulnar nerve injury, and in a few cases muscle weakness innervated by median nerve may occur. Moreover, the pyramidal signs and other related symptoms and signs of cortical involvement are often lacking. Thus, patients with hand knob stroke can easily be misdiagnosed as “peripheral neuropathy” and get the delayed or improper management [3, 4]. Until present, data on hand knob stroke are still mostly limited to case reports or small series worldwide [5–7], while only a few cases have been reported in China, which may be related to the lack of clinical awareness of the disease. Therefore，this study analyzed the clinical characteristics, stroke etiology and prognosis of 19 patients with hand knob stroke in China, in order to improve clinicians’ knowledge of the uncommon stroke at the specific site and guide the clinical practice.

## Methods

### Patients

This patients with acute stroke in the hand knob area from the Department of Neurology of the Beijing Geriatric Hospital between January 2018 and January 2022 were enrolled. The inclusion criteria were: (1) acute stroke diagnosed according to World Health Organization criteria [8], and new isolated lesion in the hand knob area or multiple lesions affected the area confirmed by brain computed tomography (CT) and cranial MRI, (2) the detailed clinical and radiological evaluations after admission and follow-up for at least 3 months. The exclusion criteria were: (1) new lesions in the hand knob area due to other causes, (2) old lesions or surgical history in the hand knob area, (3) severe neurological deficits due to previous stroke or other diseases. This study was approved by the ethics committee of the Beijing Geriatric Hospital. All patients provided written informed consent before their inclusion in the study.

### Clinical data collection

Clinical data were collected including age, gender, cerebrovascular risk factors (hypertension, diabetes, hyperlipidemia, hyperhomocysteinemia, history of stroke, heart disease, smoking, alcoholism), laboratory tests (blood cell count, coagulation function, blood biochemistry and immune function), clinical manifestations, treatment strategies, and a comprehensive cardiac screening if necessary (12-lead and 24-hour Holter electrocardiogram, transthoratic or transesophageal echocardiography and right ventricular contrast echocardiography). Finally, the etiology of hand knob stroke was determined based on the clinical data and results of ancillary examinations, and for acute ischemic stroke, the etiological classification was according to TOAST criteria including large-artery atheroscerosis (LAA), cardioembolism (CE), small-vessel occlusion (SVO), stroke of other determined etiology (SOE), and stroke of undetermined etiology (SUE) [9].

### Imaging analysis

After admission, all patients underwent the brain CT and cranial MRI, who were evaluated by magnetic resonance angiography (MRA), CT angiography (CTA) and carotid color ultrasound to complete intra-extracranial vascular assessment. Some patients were examined by high-resolution MRI (HR-MRI) and digital subtraction angiography (DSA) to determine the definitive etiology. For hand knob stroke, the size, location and distribution of new lesion were recorded, and the corresponding relationship between lesion location and finger weakness was analyzed.

### Follow-up and prognostic criteria

All patients underwent routine follow-up for at least 3 months after discharge. The clinical outcome was determined by the Modified Rankin Scale (MRS) and clinical endpoint including recurrent stroke or all-cause death in the follow-up. Finally, the MRS ≤ 2 was defined as a good prognosis, whereas the MRS>2 or occurrence of an end-point event was defined as a poor prognosis.

### Statistical analysis

All statistical analysis was performed with SPSS version 23 (IBM Corp., Armonk, NY, USA). Continuous variables were expressed as means ± standard deviation. Categorical variables were presented as frequencies and percentages.

## Results

### Demographics and baseline features

The detailed information of 19 patients with acute hand knob stroke are summarized in Table 1. There were 15 male (78.9%) and 4 female (21.1%) patients, with a mean age of 62.2 (31–83) years. These patients accounted for 0.9% of all 2224 acute stroke patients treated in our department during the same period. Seventeen patients (89.5%) were diagnosed with ischemic stroke, and 2 patients (10.5%) were hemorrhagic stroke. The cerebrovascular risk factors included hypertension (13 cases, 68.4%), hyperlipidemia (11 cases, 57.9%), hyperhomocysteine (11 cases, 57.9%), smoking (10 cases, 52.6%), alcoholism (7 cases, 36.8%), diabetes (6 cases, 31.6%), cardiovascular disease (3 cases, 15.8%: one acute myocardial infarction, one unstable angina, one left ventricular mural thrombi). All patients presented with the abrupt onset of contralateral hand paresis, including 12 cases (63.2%) of isolated hand paralysis, 4 cases (21.1%) with mild hand numbness and 3 cases (15.8%) with transient dysarthria, who had no other accompanying symptoms (headache, nausea, vomiting, etc.). The distribution of finger weakness was that radial weakness involving the thumb and index was in 3 cases (15.8%), ulnar weakness involving the third to fifth digits in 6 cases (31.6%), and uniform finger involvement in 10 cases (52.6%).

**Table 1:** Patient’s information

**Age (years, means ± SD)**	62.2 ± 13.2
**Gender (male/female)**	15/4
**Vascular risk factors**	
Hypertension	13 (68.4%)
Hyperlipidemia	11 (57.9%)
Hyperhomocysteinemia	11 (57.9%)
Smoking	10 (52.6%)
Alcoholism	7 (36.8%)
Diabetes mellitus	6 (31.6%)
Cardiac disease	3 (15.8%)
**Clinical manifestations**	
Isolated hand paresis	12 (63.2%)
Hand paresis with mild hand numbness	4 (21.1%)
Hand paresis with transient dysarthria	3 (15.8%)
**Stroke type**	
Hemorrhagic stroke	2 (10.5%)
Ischemic stroke	17 (89.5%)
**TOAST classification of cerebral infarction**	*n =* 17
SAO	6 (35.3%)
LAA	6 (35.3%)
SOE	2 (11.8%)
SUE	2 (11.8%)
CE	1 (5.9%)
**Follow-up**	
Good prognosis (mRS ≤2)	18 (94.7%)
Stroke recurrence	1 (5.3%)

*SAO* Small-vessel occlusion, *LAA* Large-artery atherosclerosis, *SOE* Stroke of other determined etiology, *SUE* Stroke of undetermined etiology, *CE* Cardioembolism, MRS Modified Rankin Scale

### Etiological analysis

The cause of hemorrhagic stroke in 2 cases was hypertension. TOAST etiological classification of 17 patients with ischemic stroke was SVO in 6 cases (35.3%), LAA in 6 cases (35.3%), SOE in 2 cases (11.8%); SUE in 2 cases (11.8%) and CE due to left ventricular aneurysm with mural thrombi in 1 case (5.9%). In the LAA subtype, the culprit vascular lesions were five ipsilateral extracranial internal carotid artery (ICA) stenosis and one ipsilateral middle cerebral artery (MCA) stenosis. Two rare causes included ICA dissection in a young male and ipsilateral ICA occlusion due to compression of carotid body tumor in an elderly male.

### Imaging features in hand knob stroke

**Fig. 1:**
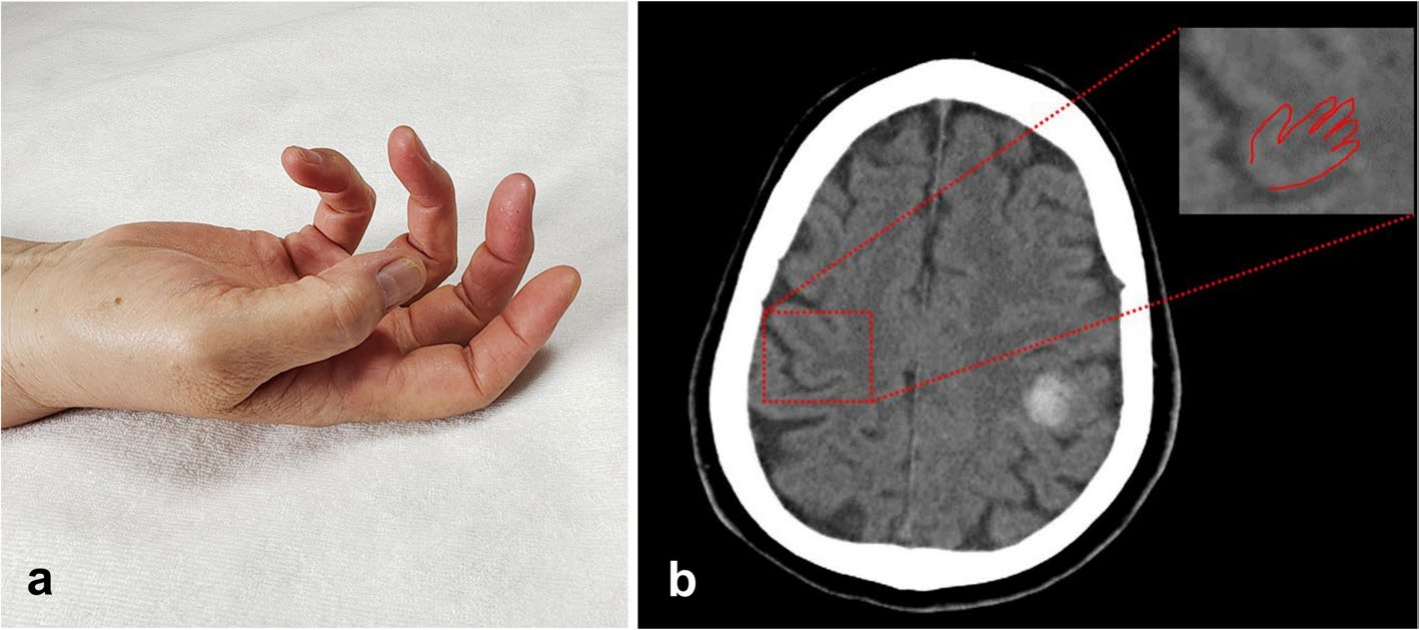
**a** 79-year-old man with a previous history of severe hypertension presented with acute isolated total hand weakness; **b** Cranial CT showed cerebral hemorrhage in the left knob area

**Fig. 2:**
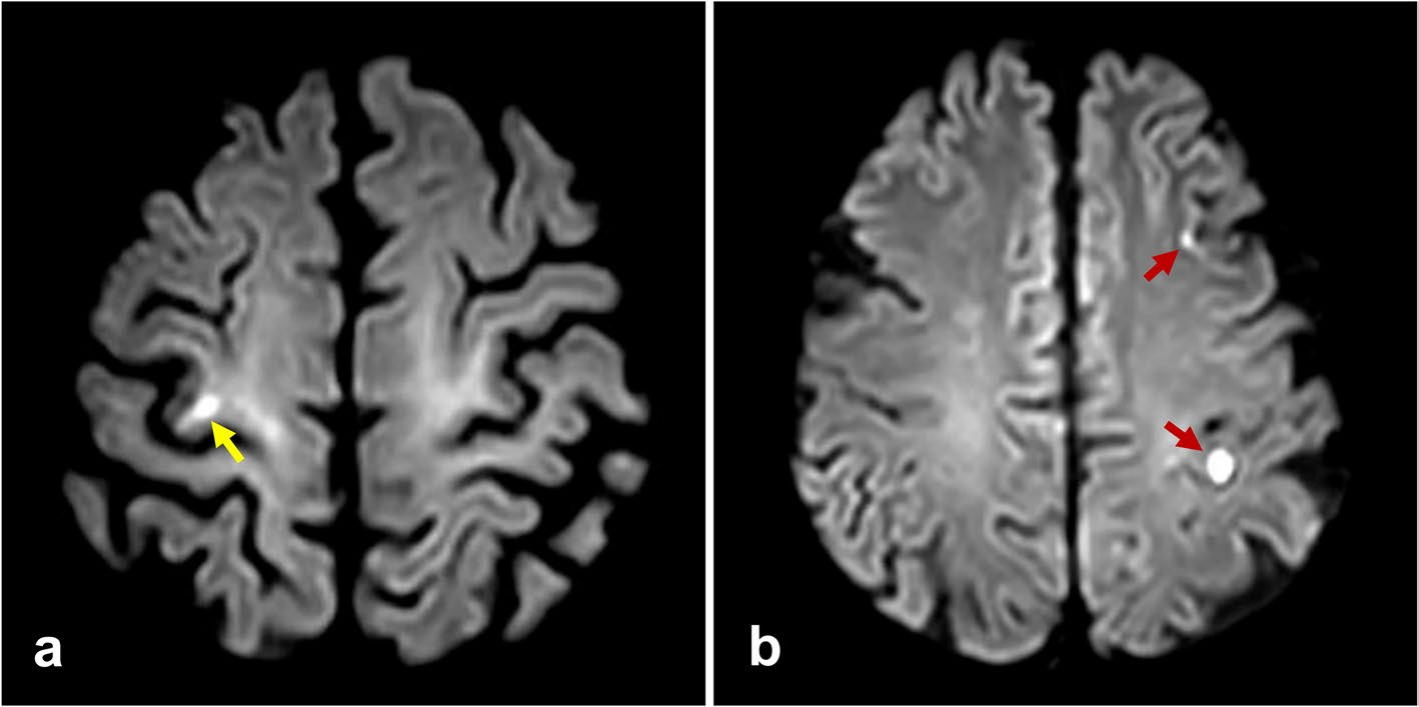
**a** DWI showed the isolated infarct located in the right knob area (yellow arrow); **b** DWI in another patient showed the multiple infarcts scattered in the left knob area and left frontal cortex (red arrows)

The lesions of two hand knob hemorrhagic stroke were round-like with the diameter less than 15 mm and only involved the hand knob area on CT (Fig. 1). In 17 cases with ischemic stroke, the DWI sequence of brain MRI showed new lesions with the diameter of 0.5-3 mm, located in the hand knob area (8 cases) or dispersed in the cortical territory of the MCA (9 cases) (Fig. 2). The location of the lesion in the hand knob area and the corresponding distribution of finger weakness was as follows: 6 cases (31.6%) with medial lesion showed ulnar finger weakness; 3 cases (15.8%) with lateral lesion showed radial finger weakness; 3 cases (15.8%) with middle lesion and 7 cases (36.8%) with large lesion involved the total area showed uniform finger weakness.

### Treatment and follow-up

The patients with LAA, SVO and SUE etiological subtypes received the long-term medical treatment of antiplatelet and control of risk factors, and two patients with the LAA etiological subtype also underwent carotid stenting. The patient with carotid body tumor was treated with conventional secondary prevention of cerebrovascular disease, and the vascular surgeon recommended regular follow-up of tumor size changes; The patient with carotid artery dissection was treated oral anticoagulant for 1 year without recurrence and then discontinued. The patient with CE was given long-term oral anticoagulant therapy. Two patients with cerebral hemorrhage were treated conservatively.

The median follow-up period was 13.5 months (3–29 months) in this study. Eighteen patients (94.7%) had a favorable outcome during the follow-up, whereas one patient (5.3%) with severe ICA stenosis who refused endovascular treatment at first hospitalization developed recurrent stroke 7 months after discharge, and there was no any further stroke recurrence after carotid stenting.

## Discussion

To the best of our knowledge, in this study we presented the largest cohort of patients with hand knob stroke in mainland China and summarized the clinical features, etiology, and prognosis.

Hand knob stroke is often considered an uncommon type of stroke with an incidence rate less than 1% [6, 10]. In the present study, hand knob stroke accounted for 0.9% of all acute strokes, which was consistent with previous reports. The low diagnostic rate may be due to lack of recognition of hand knob stroke by some physicians, who misdiagnosed hand knob stroke as peripheral neuropathy because of the similar symptoms, but it may also be attributed to the decrease in the visit rate as a result of a mild clinical symptom and a good recovery of limb function [6, 7]. In addition, the detection rate may also be impacted if the isolated lesion in the hand knob area is minor and the MRI is not performed promptly. Wang Y et al [11] reported that hyperhomocysteinemia was the most common risk factor for hand knob infarction, while Orosz P et al [7] considered that hypertension was the most common. Our results showed that hypertension was the most frequent risk factor in patients with hand knob stroke, followed by hyperlipidemia and hyperhomocysteinaemia. The difference may be attributed to the different sample size, ethnicity and environment. In this cohort, there were more male subjects than female, and compared with the previous reported literature, the cohort had a larger age span (ranged from 31 to 83 years).

In this study, 12 of 19 patients presented isolated hand paresis without pyramidal signs or other cortex-related signs of anterior circulation. Although hand knob stroke can be easily misdiagnosed as the peripheral neuropathy, a careful physical examination revealed that the distribution of weak fingers did not follow the pattern of peripheral nerve distribution [12, 13]. This study found that the medial lesion showed ulnar fingers weakness, the lateral lesion showed radial fingers weakness, the middle or the large lesion involving the whole hand knob area showed homogeneous finger weakness. The possible reason was that different parts of hand knob area may have the corresponding somatotopic representation of fingers [14, 15].

Almost all of the causes of hand knob stroke reported at home and abroad were ischemia. In this study, we reported for the first time two cases related to cerebral hemorrhage, which extended the current knowledge on the aetiology of hand knob stroke. These two lesions of intracerebral hemorrhage were confined to the hand knob area, which were classified as lacunar stroke due to the small size. Arboix A et al [16] analyzed 17 patients with hemorrhagic lacunar stroke and the results implied that hypertension was the primary cause and the patients had the favorable outcome, which was consistent with our results. Studies on the etiology and pathogenesis of hand knob infarction had been inconsistent in the literatures. A total of 15 cases were included in the study by Alstadhaug KB et al [17], 77% of whom had radiological findings of ischemic white matter lesions, so the authors believed that cerebral small vessel disease was the main etiology in the hand knob infarction. Castaldo J et al [5] selected 35 cases for analysis and found that CE (46%) was the main etiological type, followed by LAA (34%), while other studies considered that the main mechanism for hand knob infarction was arterio-arterial embolism based on LAA [7, 10]. In the present study, SVO (35.3%) and LAA (35.3%) were the main causes of hand knob infarction. Of note, all 6 patients with LAA had multiple lesions close to the cortex, and 5 of whom exhibited ipsilateral ICA stenosis, indicating a possible arterio-arterial embolic mechanism for LAA patients, which was consistent with the previous reports [10]. In one patient with CE, the embolus came from the left ventricle, reminding that it is necessary to perform a thorough cardiac examination. In previous studies, rare causes of hand knob infarction included moyamoya disease [11], giant cell arteritis [18], cancer-associated thromboembolism [19]. In this cohort, carotid dissection and carotid body tumor were reported for the first time as the rare causes of hand knob stroke.

There were few studies on the prognosis of hand knob stroke, and two previous studies with long-term follow-up of hand knob stroke (median 25 and 30 months) had shown good long-term clinical outcomes [5, 20]. In this study, the majority of patients (94.7%) had also a good prognosis during a median follow-up period of 13.5 months. Some investigators considered that the favorable prognosis may be related to abundant collateral blood supply in the hand knob area and functional compensation in other areas [21, 22]. In the cases with poor prognosis, Peters N et al [10] reported that 2 patients (6%) with hand knob stroke developed acute myocardial infarction and 1 (3%) had recurrent cerebral infarction during follow-up, while Alstadhaug KB et al [17] reported that 2 patients (15%) died of cardiac arrest and sudden unexplained death during follow-up. Notably, one patient (5.3%) with severe stenosis of bilateral ICA had recurrent stroke due to refusal of the interventional therapy at first hospitalization in this study. All in all, although the overall prognosis of hand knob stroke is good, non-pharmacological approaches are likely necessary to reduce the stroke recurrence of these patients with LAA etiology. The hand knob stroke may have potentially severe vascular lesions or complicated stroke mechanisms, so such patients should not be overlooked due to mild symptoms. Thus, the further long-term follow-up studies are warranted to elucidate the natural course and factors affecting prognosis of the hand knob stroke.

## Conclusions

Hand knob stroke is an uncommon type of stroke with a good prognosis and a low recurrence rate. Ischemic stroke is the predominant type of hand knob stroke, and the main clinical manifestation is contralateral hand paresis, and the topography of the lesion corresponds to finger weakness. The cause of hand knob hemorrhage is hypertensive, SVO and LAA are the main causes of hand knob infarction, but there are some rare etiologies. The limitations of this study are that the number of cases is still small, some patients have the short follow-up time, and different treatments for different etiologies may also affect the prognosis. Therefore, further prospective studies with a large sample size are expected to confirm these results.

## Data Availability

The dataset(s) used and/or analyzed during the current study are available from the corresponding author on reasonable request.

## Abbreviations

MRI: Magnetic resonance imaging
CT: Computed tomography
LAA: Large-artery atherosclerosis
CE: Cardioembolism
SAO: Small-vessel occlusion
SOE: Stroke of other determined etiology
SUE: Stroke of undetermined etiology
MRA: Magnetic resonance angiography
CTA: Computed tomography angiography
HR-MRI: High-resolution magnetic resonance imaging
DSA: Digital subtraction angiography
DWI: Diffusion-weighted imaging
MRS: Modified Rankin Scale
ICA: Internal carotid artery
MCA: Middle cerebral artery

## References

[1] Yousry TA, Schmid UD, Alkadhi H, Schmidt D, Peraud A, Buettner A (1997). Localization of the motor hand area to a knob on the precentral gyrus. A new landmark. Brain.

[2] Celebisoy M, Ozdemirkiran T, Tokucoglu F, Kaplangi DN, Arici S (2007). Isolated hand palsy due to cortical infarction: localization of the motor hand area. Neurologist..

[3] Phan TG, Evans BA, Huston J (2000). Pseudoulnar palsy from a small infarct of the precentral knob. Neurology..

[4] Tahir H, Daruwalla V, Meisel J, Kodsi SE (2016). Pseudoradial nerve palsy caused by acute ischemic stroke. J Investig Med High Impact Case Rep.

[5] Castaldo J, Rodgers J, Rae-Grant A, Barbour P, Jenny D (2003). Diagnosis and neuroimaging of acute stroke producing distal arm monoparesis. J Stroke Cerebrovasc Dis.

[6] Finkelsteyn AM, Saucedo MA, Miquelini LA, Chertcoff A, Bandeo L, Pacha S (2019). Ischemic stroke of the "hand knob area": a case series and literature review. J Clin Neurosci.

[7] Orosz P, Szőcs I, Rudas G, Folyovich A, Bereczki D, Vastagh I (2018). Cortical hand knob stroke: report of 25 cases. J Stroke Cerebrovasc Dis.

[8] Stroke--1989. Recommendations on stroke prevention, diagnosis, and therapy. Report of the WHO task force on stroke and other cerebrovascular disorders. Stroke. 1989;20(10):1407–31. 10.1161/01.str.20.10.1407 2799873

[9] Adams HP, Bendixen BH, Kappelle LJ, Biller J, Love BB, Gordon DL (1993). Classification of subtype of acute ischemic stroke. Definitions for use in a multicenter clinical trial. TOAST. Trial of org 10172 in acute stroke treatment. Stroke..

[10] Peters N, Müller-Schunk S, Freilinger T, Düring M, Pfefferkorn T, Dichgans M (2009). Ischemic stroke of the cortical "hand knob" area: stroke mechanisms and prognosis. J Neurol.

[11] Wang Y, Dong Q, Li SJ, Hu WL (2018). New clinical characteristics and risk factors of hand knob infarction. Neurol Sci.

[12] Brigo F, Ragnedda G, Canu P, Nardone R (2018). Synkinetic wrist extension in distinguishing cortical hand from radial nerve palsy. Pract Neurol.

[13] Kim JS (2001). Predominant involvement of a particular group of fingers due to small, cortical infarction. Neurology..

[14] de Medeiros FC, Viana DCR, Cunha MN, Hatasa CC, Araújo RV (2017). Pure motor monoparesis due to infarction of the "hand knob" area: radiological and morphological features. Neurol Sci.

[15] Gass A, Szabo K, Behrens S, Rossmanith C, Hennerici M (2001). A diffusion-weighted MRI study of acute ischemic distal arm paresis. Neurology..

[16] Arboix A, García-Eroles L, Massons J, Oliveres M, Targa C (2000). Hemorrhagic lacunar stroke. Cerebrovasc Dis.

[17] Alstadhaug KB, Sjulstad A (2013). Isolated hand paresis: a case series. Cerebrovasc Dis Extra.

[18] Shelley BP, Harishchandra P, Devadas AK (2020). Selective hand motor cortex lesions masquerading as "Pseudoperipheral nerve palsy". Ann Indian Acad Neurol.

[19] Tomoda Y, Tanaka M, Tanaka K (2019). Hand knob stroke from cancer-associated thromboembolism. CMAJ..

[20] Wardlaw JM, Smith EE, Biessels GJ, Cordonnier C, Fazekas F, Frayne R (2013). Neuroimaging standards for research into small vessel disease and its contribution to ageing and neurodegeneration. Lancet Neurol.

[21] Kesserwani H (2020). Ischemic infarct of the hand knob gyrus: natural history, morphology, and localizing value of the omega sulcus - a case report with a side note on the dynamic forces underlying sulci formation. Cureus..

[22] Willett FR, Deo DR, Avansino DT, Rezaii P, Hochberg LR, Henderson JM (2020). Hand knob area of premotor cortex represents the whole body in a compositional way. Cell..

